# Review of Advances in Fire Extinguishing Based on Computer Vision Applications: Methods, Challenges, and Future Directions

**DOI:** 10.3390/s25206399

**Published:** 2025-10-16

**Authors:** Valentyna Loboichenko, Grzegorz Wilk-Jakubowski, Lukasz Pawlik, Jacek Lukasz Wilk-Jakubowski, Roman Shevchenko, Olga Shevchenko, Radoslaw Harabin, Artur Kuchcinski, Valentyna Fedorchuk-Moroz, Anastasiia Khmyrova, Ivan Rushchak

**Affiliations:** 1 Departamento de Ingeniería Energética, Escuela Técnica Superior de Ingeniería, Universidad de Sevilla, Camino de los Descubrimientos s/n, 41092 Sevilla, Spain; 2 Department of Civil Security, Lutsk National Technical University, Lvivska St., 75, 43000 Lutsk, Ukraine; fedmor70@ukr.net; 3 Institute of Internal Security, Old Polish University of Applied Sciences, 49 Ponurego Piwnika Str., 25-666 Kielce, Poland; grzegorzwilkjakubowski@wp.pl (G.W.-J.); r.harabin@stsw.edu.pl (R.H.); akuchcinski@stans.edu.pl (A.K.); 4 Institute of Crisis Management and Computer Modelling, 28-100 Busko-Zdrój, Poland; 5 Department of Information Systems, Kielce University of Technology, 7 Tysiąclecia Państwa Polskiego Ave., 25-314 Kielce, Poland; lpawlik@tu.kielce.pl; 6 Science and Innovation Center, National University of Civil Defence of Ukraine, 8 Onoprienka Str., 18034 Cherkasy, Ukraine; shevchenko605@i.ua (R.S.); shevchenkoolga2008@gmail.com (O.S.); khmyrova.anast@gmail.com (A.K.); rushchak_31@ukr.net (I.R.)

**Keywords:** firefighting robots, fire detection, fire extinguishing, aerial vehicles, computer vision, fire suppression, crisis management, safety

## Abstract

This paper examines the state-of-the-art in fire suppression technologies based on computer vision applications in the subject areas of computer science and engineering. The study involves a two-stage analysis of publications using keywords. This paper presents a bibliographic analysis of scientific literature from the Scopus database using VOSviewer software and the author’s methodological approach. General keywords were used for the initial analysis of the dataset, followed by a more detailed study with additional criteria and specific keywords. The categories considered in the article are as follows: Firefighting Robots, Fire Detection, Fire Suppression, Aerial Vehicles, and Computer Vision. It is shown that the research includes technical aspects of fire robots and systems, as well as the improvement of their software and hardware. The subsequent review highlights the important role of computer vision in improving the efficiency and effectiveness of fire suppression systems. It is noted that key advances include the development of sophisticated fire detection algorithms and the implementation of automated fire suppression systems. The study also discusses the challenges and future directions in this field, emphasizing the need for continuous innovation and interdisciplinary collaboration. This review provides valuable information for researchers, engineers, and practitioners in the field of fire safety by offering a comprehensive overview of state-of-the-art technologies and their applications in fire suppression.

## 1. Introduction

In practice, scientific efforts in numerous academic and research centers around the world are directed toward ensuring broadly understood safety, which can take various forms depending on the location of the activities (on land, at sea, or in the air) [1,2,3,4,5,6,7,8,9,10]. One of the natural and civilizational threats is fire, which constantly endangers the lives of humans and animals, and through the devastation of the environment and extensive destruction, causes enormous losses in environmental, infrastructural, and economic terms [11,12,13,14]. The psychological (mental) aspect is also of considerable importance—people may suffer from psychological issues and trauma as a consequence of fires [15,16]. Since these phenomena can be caused by both natural factors and human activities, and cannot be entirely prevented, innovative methods for flame detection and fire suppression are constantly being sought [10,15,16,17,18,19].

**Figure 1:**
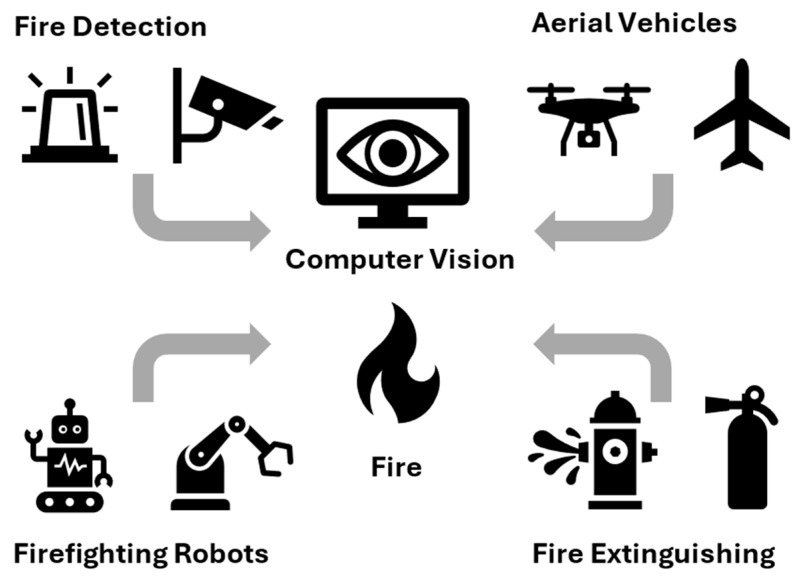
Interconnections between different elements in fire management.

From a practical point of view, fire management involves several aspects, such as flame detection and extinguishing (see Figure 1). Fire management techniques [10,18,19], combined with recent advances in scientific and technological thought—including artificial intelligence algorithms for multifaceted image processing aimed at fire detection and monitoring—can contribute to a better understanding of the causes of fires, the phenomena accompanying them, detecting flames at significantly greater distances than traditional smoke or temperature sensors, and—once a fire is positively detected—determining the direction of flame spread and enabling rapid extinguishing [20,21]. This is particularly important given that environmental parameters and weather fluctuations affect the performance of such systems [22,23,24].

Detection becomes possible not only through visual observation, but also through the use of computer vision with monitoring cameras as a supervisory element. When flames are detected, alarm sirens can be activated to signal the event, and appropriate firefighting services are dispatched to the scene. Computer vision enables remote and safe identification of fire indicators (e.g., detection of smoke or flames) [20,21,22,25,26], using aerial vehicles, including drones, especially in open areas. Their use significantly extends the monitoring range compared to traditional solutions. This is particularly useful for estimating the size of fires in hard-to-reach areas that are cut off from the outside world. Modern integrated systems enable automated and rapid responses to fire hazards, contributing to faster flame suppression and, consequently, reducing both material and non-material losses resulting from fires.

On the other hand, the growth in the number of scientific journals and the increase in publications in various areas of research lead to the emergence of a significant amount of information and materials, which complicates the work of scientists in systematizing data and identifying trends and unresolved issues in certain areas. As a result, there is a development of the direction of bibliographic analysis, when research is directly related to the analysis of the state of the issue in the scientific literature, including using various scientific databases (Scopus, Web of Science, PubMed, etc.) and specialized software such as VOSviewer [27].

For example, bibliometric analysis by the authors of short-term incomprehensible events and life-threatening events [28], evacuation lighting of the disaster site [29], the role of social media and platforms in natural super-emergency situations when mobile communication devices do not work [30], determining the safety and stability of systems and communities at the current level of technology [31], postoperative behavioral disorders of patients [32], waste management, etc. In the field of fire safety, bibliometric analysis is used to determine the main scientific directions [33], trends in IoT-enabled fire safety systems [34], methods for fire evacuation training in buildings [35], and fire prevention and mitigation technologies in high-rise buildings [36], etc.

Thus, the aim of the work is to study the current state of the issue in the field of fire extinguishing based on computer vision applications using bibliometric analysis approaches.

This review paper presents recent advances in fire extinguishing based on computer vision applications. The methodology, including the adopted research framework, will be described at the outset. Then, a general review of the scientific literature from the Scopus database will be presented, including using the VOSviewer software [27]. Then, based on data from the Scopus database covering the years 2015–2024, the state-of-the-art will be presented using both qualitative approach and quantitative approaches. The analysis will take into account three main areas (which will be understood in the further part of the paper as main categories or domains): Firefighting Technology, including the research directions undertaken by the authors, the methodology applied (i.e., Research Methodology), and Document Type.

On this basis, it is possible not only to identify the latest research directions, taking into account the research methodology and types of publications (summary of the state-of-the-art based on the publisher-independent Scopus database), but also to assess whether there is a relationship between the variables analyzed using the chi-square test (χ^2^). The quantitative results obtained through the applied methodological approach are complemented by chi-square statistics, which enable the verification of whether there are statistically significant or non-statistically significant changes in the analyzed data, representing a scientific novelty in this area. Furthermore, the work presents a brief analysis of the main content of the categories identified by the authors, noting unresolved problems.

## 2. Materials and Methods

### 2.1. The Categories Applied

To identify publications from the Scopus database that meet thematic criteria, a descriptive and comparative method, as well as elements of system analysis, was applied. The selection of appropriate categories was preceded by an exploration of the Scopus database, focusing on publications in which specific words appear in the title, abstract, and keywords of the publication, according to the following scheme:

(TITLE-ABS-KEY (“Fire Extinguishing” OR “Fire Fighting”) AND TITLE-ABS-KEY (“Aircraft”) OR TITLE-ABS-KEY (“Computer Vision”) OR TITLE-ABS-KEY (“Fire Detectors”) OR TITLE-ABS-KEY (“Extinguishing System”) OR TITLE-ABS-KEY (“Pattern Recognition”) OR TITLE-ABS-KEY (“Robot”)).

On this basis, 1994 publications were obtained and subjected to further analysis (at 27 March 2025). This phase involved a general analysis of the literature available in the Scopus database and the use of the VOSviewer (v. 1.6.20) software to identify common features and trends. VOSviewer is an open-source software for bibliometric analysis of literature, detection of co-occurrence, co-authorship, and co-citation, and the construction of corresponding maps for visualization of the obtained results. In the work, a series of maps was constructed in the co-occurrence mode for all keywords that occur at least 10 times, including their distribution by clusters, frequency of use, and density of use over time.

**Figure 2:**
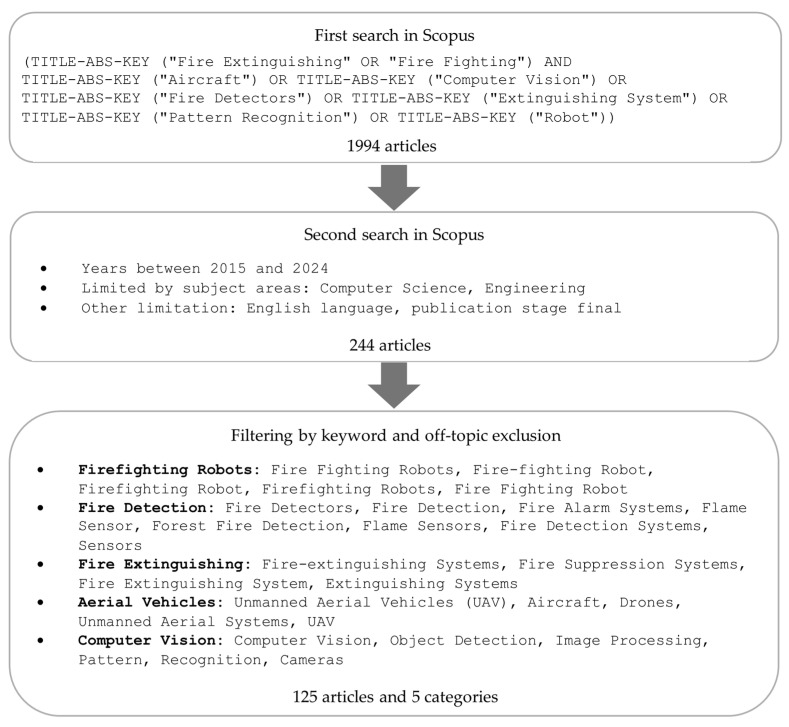
Scheme for screening and preparing data for further analysis.

The publications identified were limited to those related to Computer Science and Engineering as subject areas, had a status of completion (final stage), and were published between 2015 and 2024 in English, in line with the objective of identifying the latest research trends. For the 244 publications identified, significant keywords were selected using Scopus, and the articles were grouped into relevant categories. Each publication was then screened, and those that did not apply were manually eliminated: Emotion Recognition, Warehouse Design, Fiducial Marker Detection, Pedestrian Detection, Radio Communications, Seawater Fog, Autonomous Ships, and Detection System on Ships. As a result, 125 publications that met the established criteria were obtained and included for further analysis. The detailed data exploration process is presented in Figure 2.

In practice, all publications found in the Scopus database were subjected to analysis to delineate one overarching main area related to Firefighting Technology. Within this domain, five subcategories (subdomains) were specified: Firefighting Robots, Fire Detection, Fire Extinguishing, Aerial Vehicles, and Computer Vision. The publications that belonged to the Firefighting Technology domain contain, in particular, (1) various types and purposes firefighting robots; (2) Fire Detection, including fire detector systems, fire alarm systems, and flame and smoke sensors; (3) Fire Extinguishing—including fire extinguishing and suppression systems; (4) Aerial Vehicles—including unmanned aerial vehicles (UAV), aircrafts, and drones; (5) Computer Vision—including object detection, image processing, pattern, recognition and cameras.

In this paper, a distinction is made between the classification of documents assigned to one of the three main types of publications (i.e., Document Type). The first one is the conference paper, the second is the journal article, and the last is another category which may include, among other things, reviews of books and chapters, conference reviews, scientific works, letters, errata, editorials, notes, short surveys, and data papers. When analyzing documents from the Scopus database, the research methodology applied is considered. According to the methods used in research, each publication is assigned to the appropriate subcategory: (1) Experimental (laboratory and field); (2) Literature Analysis (including textual and legal source analysis applying semiotic analysis to books, book chapters, academic papers, etc.); (3) Case Study (with field-derived reports); and (4) Conceptual (focuses on developing and refining new theories and concepts).

It should be noted that, depending on the subject matter covered and the methods used for research, each document was analyzed to belong to multiple categories and subcategories. In practice, this allows for a multi-label classification, where a single document can belong to more than one research category. The same principle applies to research methods and countries (if several methods are applied, they are all assigned to the document, as shown in the Results and Analysis section). In the case of countries, the quantitative analysis focused on the countries with the highest publication activity, while other countries were included in the subcategory Other.

The features of qualitative (thematic) analysis, based on state-of-the-art research from the last ten years, are presented in Section 2.2.

### 2.2. Recent Developments: An Overview from 2015 to 2024

As previously indicated, within the main category Firefighting Technology, five subcategories were identified, each understood as a subdomain. Based on this classification, the manuscript describes the main blocks, which, alongside Research Methodology and Document Type, will be subjected to detailed quantitative analysis in the next section (i.e., Results and Analysis).

## 3. Results and Analysis

### 3.1. General Review of Publications Related to the Firefighting Technology in the Scopus Database

**Figure 3:**
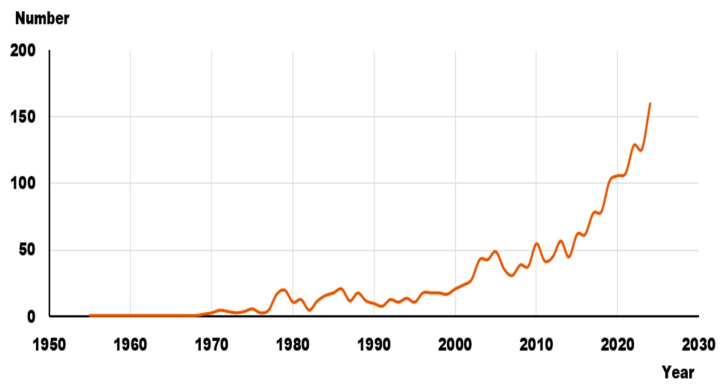
Dependence of the number of publications on the researched issue on the year of publication.

According to the presented methodology, a primary analysis of scientific publications presented in the Scopus database in the area of study was conducted. Figure 3 shows the dynamics of the dependence of the number of publications on the year of publication. As can be seen, before 2000, there was no significant number of scientific publications in the area under study, whereas since 2005, there has been an increase in scientific interest. And in the last 10 years, a sharp surge in publications can be noted (from 50 publications per year to 150).

**Figure 4:**
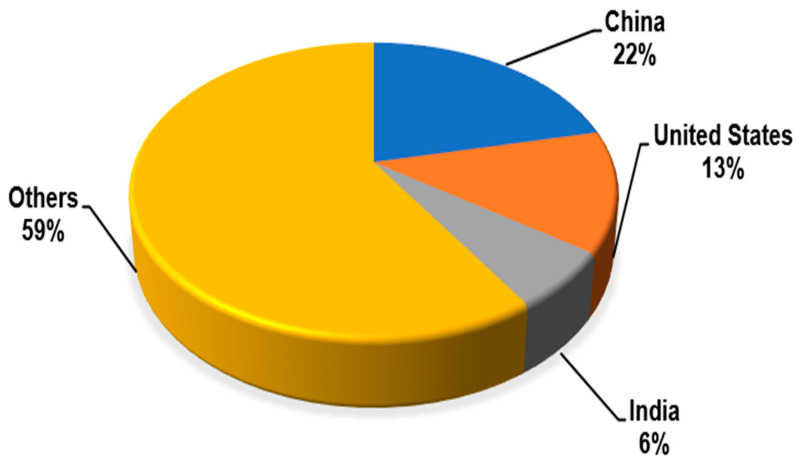
Distribution of the number of publications on the research question by country.

An analysis of the number of publications by country (see Figure 4) shows that China is the leader (1/5 of all publications), followed by the United States, and third place goes to India. The remaining countries provide almost 60% of publications.

As can be seen from the data presented in Table 1, the leading organization in the study of fire extinguishing issues in the direction indicated in this work is the University of Science and Technology of China. The second and third positions, with an equal number of works, are distributed between authors affiliated with the Ministry of Education of the People’s Republic of China and Technion–Israel Institute of Technology. Trinity College Hartford and the National Institute of Standards and Technology, located in the USA, are affiliated with 18 and 16 publications, respectively.

An analysis of the works, taking into account the author’s contribution, showed (Table 2) that the system cannot identify some authors for various reasons (the so-called “Anon”). The leading experts in terms of the number of publications are Ahlgren D. (Trinity College, Hartford, U.S.) and Verner I. (Technion–Israel Institute of Technology).

**Figure 5:**
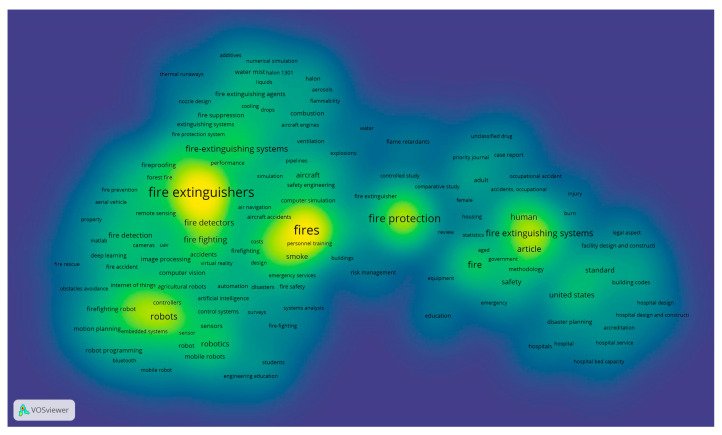
Map of keyword density in publications generated using the VOSviewer program.

Further research into the issue involved the use of VOSviewer software. As can be seen from Figure 5, the visualization of 1994 publications (in the “co-occurrence” mode) shows the most frequent use of such words as “Fire extinguishers”, “Fires”, “Fire protection”, “Robots”, and “Fire extinguishing systems”. This may indicate the relevance of the technical component of fire extinguishing and the tendency to explore the possibility of robotizing (automating) processes.

**Figure 6:**
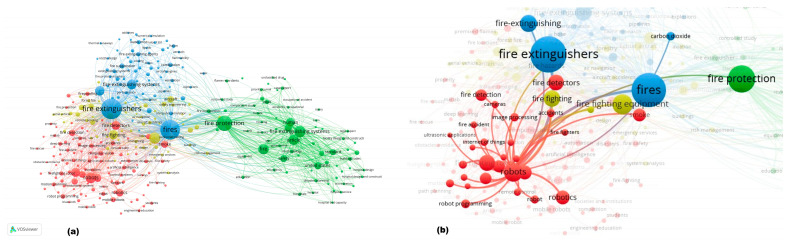
Map visualization of the keyword network, with distribution by clusters generated using the VOSviewer program: (**a**) general view; (**b**) highlighted connections for the cluster “Robots”.

Network Visualization of the data (Figure 6a) shows the distribution of keywords across 4 clusters: “Fire extinguishers”, “Fire protection”, “Robots”, and “Fire fighting equipment” (the size of the mark with the word determines the frequency of occurrence, its “weight”). The “Fire extinguishers” cluster, like the “Fire fighting equipment” cluster, has two distinct cores (“Fire extinguishers”-“Fire” and “Fire fighting equipment” - “Fire fighting”). Thus, according to the analysis of relationships (Figure 6a), studies related to “Fire” are evenly distributed among the other three clusters. In contrast, the “Fire extinguishers” core has more relationships with the “Robots” and “Fire fighting equipment” clusters, highlighting the diversity of research areas. The “Fire fighting equipment” core is associated with a large number of keywords in the “Fire protection” and “Robots” clusters, while the “Fire fighting” core is mainly associated with the “Robots” cluster. The “Fire protection” keyword, as can be seen from Figure 6a, is located in a separate cluster, although it is closely related to the “Fire extinguishers” cluster and the “Fire” core. At the same time, for the cluster “Robots” (Figure 6b), with an obvious thematic relationship with “Fire”, a direct connection with such areas as equipment (“Fire extinguishers”, “Fire fighting equipment”), protection (“Fire protection”), and fire detection (“Fire detection”) is also noted.

**Figure 7:**
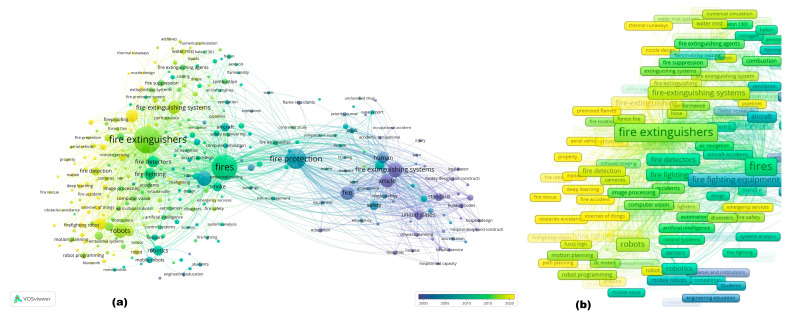
The map of keyword co-occurrence by year (2020–2025) was generated using the VOSviewer program: (**a**) general view; (**b**) enlarged view of publications in recent years.

When studying the distribution of keywords co-occurrence by year (Figure 7a), the research on issues directly related to fire extinguishing systems, incidents, health aspects, standardization of fire extinguishing, fires, and design features (before 2000) gradually shifted towards “Fire protection” (2000–2005). Then, the development of fire extinguishing technologies led to a more in-depth study of issues directly related to fire, fire combustion, gaseous fire extinguishing agents, fire safety, risks, fires in aircraft, and simulations (2005–2015). The next 5 years (2015–2020) were significantly associated with “Fire extinguishers”, detection, robots, fire extinguishing agents, and the development of fire extinguishing systems.

At the same time, research is beginning to appear on computer vision, artificial intelligence, control systems, mobile robots, and programming. In modern research, there is a general trend toward a shift in research interest toward deep learning, the Internet of Things, robots, and fire extinguishers (Figure 7b). Thus, we can note an intensification of research into the potential of digital technologies, artificial intelligence, and robotics in firefighting technologies, which is consistent with the stated Sustainable Development Goals and reflects the current transformation of society as part of the transition to Industry 5.0.

Given the increased interest in the research area of Firefighting Technology over the past 10 years, it is appropriate to conduct a more detailed analysis of these publications according to the approach proposed by the authors.

### 3.2. Thematic Review of Publications Related to the Firefighting Technology Category (2015–2024)

Below is a thematic review of publications related to the Firefighting Technology category from a qualitative perspective (i.e., taking into account the research directions pursued by researchers).

In practice, fire poses a significant threat to life and property in every society, while shared traditions of firefighting services and crisis management systems have evolved over centuries [37]. The urban planning aspect plays a crucial role in preparedness, particularly the location of fire stations within a given area, taking into account fire risk [38]. Fire-related losses can be minimized through the effective prevention of fire spread and by enabling safe and rapid evacuation. This becomes possible with the implementation of dedicated flame monitoring and suppression systems, such as the T-Fire system [39]. Computer vision also plays an increasing role in fire protection, as it finds ever more applications in this field [17,40,41]. Deep learning methods can be used for quantifying firepower by flame pictures, which can help explain the functioning of vision-based mechanisms. In practice, the use of artificial intelligence techniques, including computer vision, shows potential not only for object recognition in outdoor environments but also in indoor spaces [42,43]. Robotics achievements are also of great importance, which justifies the use of robots in medicine, rehabilitation, various industrial sectors, and rescue operations, where robots often replace humans in performing tasks related to safety control [22,44,45,46,47,48,49,50,51,52,53,54,55,56,57]. From a practical point of view, fires can now be extinguished using more or less sophisticated methods [10,58,59,60,61,62]. The use of robots, in addition to enhancing safety (as it is not a human firefighter on the front line of fire combat) [15,26,63], also offers benefits in maintaining isolation between firefighters—an aspect that proved particularly important during the spread of COVID-19 infections [64]. When a fire broke out at Paris’s Notre-Dame Cathedral on April 19, 2019, firefighters, overwhelmed by the scale of the building and the fire, decided (for safety reasons) to deploy recent technological advancements directly beneath the burning roof of the cathedral: an automated rescue and firefighting robot system equipped with multiple modern technologies [65]. Currently designed robots can have multiple degrees of freedom, which, thanks to the use of proximity sensors and flame detectors, helps reduce the risk of exposing operators to fire and smoke [25]. Additionally, some of the implemented solutions provide rescue services with information from the fire scene, such as the number of human victims, their condition and location, as well as the type of fuel sustaining the fire (the best models are then used to support the first responders) [66,67].

In practice, the verification of robotic applications remains a constant challenge due to their hybrid nature and distributed architecture [68]. The use of firefighting robots equipped with integrated thermal cameras and LiDAR sensors (a fusion-based approach) enables the acquisition of valuable information from fire environments [69,70]. In modern systems, various technologies are applied, depending on the application, including infrared, Bluetooth, Wi-Fi (Wireless Fidelity), GPS (Global Positioning System), GSM (Global System for Mobile Communications), and remote sensing, e.g., [45,67,69,70,71,72,73,74,75,76,77]. Modern robots, besides detecting and identifying obstacles and temperature spikes, can also detect gases [15,47], smoke [78], and monitor air quality [79]. A recent innovation involves developing firefighting systems capable of both detecting and extinguishing flames [15,41,80,81,82,83,84]. One example is an acoustic technique in which an artificial-intelligence module detects spot fires that can then be suppressed using suitably generated and modulated acoustic waves (depending on the flame’s source) [10,19,20,21,58,60,61,62]. Such systems could find potential applications, among other things, in modes of transport where the waveguide length is advantageous.

Various optimization algorithms are implemented in autonomous robots for route planning, including the A∗ algorithm, which is suitable for planning the shortest path [85,86]. During operation, many factors influence the robot’s performance (including approaching obstacles, inflection points, redundant points, and total turning angle), which is why researchers are currently focusing their efforts on modifying traditional algorithms, including the use of the Floyd algorithm [87]. Clustering, sensor sleep/activity plan, and robot energy harvesting/moving modes are used to extend and maximize the lifespan of sensors/robots in a wireless sensor and actor networks (WSAN) [88]. Another extremely important issue is ensuring the optimal positioning of firefighting robots in the building in order to increase the coverage area of the building’s floors, which translates into minimizing the response time to a potential threat. In complex systems, routing can be based on determining the shortest path for each firefighting robot, e.g., using a fuzzy Q-learning (FQL)-based trajectory mechanism [88]. Another example can be an intelligent autopilot fire extinguishing robot (AFER) [74]. The body of this compact robot is made of heat-resistant materials. A temperature sensor continuously monitors ambient temperature; if a threshold is exceeded, a flame sensor is activated. Robots can also be equipped with night vision cameras and various types of propulsion systems [71,89,90]. The remotely controllable aerial-hose-type fire-fighting robot, which was presented during the World Robot Summit 2020 (WRS 2020) opening ceremony in Fukushima, is described in [91]. Worldwide, new structural materials continue to be sought for a variety of applications [92,93,94,95]. Some robots are equipped with a double waterproof stainless-steel casing that protects the device from flames, with additional layers of protection—especially for electronics, controllers, sensors, communications, and power systems [96,97,98,99]. Their intended use includes extinguishing fires in petrochemical plants [100,101]. Other robots are more versatile and can be used both in industrial facilities and in homes [17,102,103,104]. Undoubtedly, the application of robotics and control systems enhances the precision and ease of operating the robot [18,25,56,105,106,107,108,109]. The PSO (Particle Swarm Optimization) algorithm enables problem optimization through iterative processing [85,110]. Many researchers are also exploring adaptive control using various techniques, including the recursive backstepping method and filter banks [111,112]. Some solutions use image pre-processing algorithms (to filter areas with a likelihood of flame presence), morphological operations like dilation and erosion (using filters), and algorithms that incorporate attention mechanisms for flame recognition in processed images [113]. Mathematical methods, including spatial regression analysis and Bayesian estimation models, are also employed in analytical processes [6,7,114,115]. In practice, robots can apply deep learning not only for fire detection and classification (based on training data) but also to extinguish detected fires based on their classification [44]. The implemented visual recognition system plays a key role in effective flame detection [113]. An example of integrating a knowledge graph with a large language model (LLM) is described in [116]. A suggestion module, through generated prompts, supports the language model, which in turn assists the robot in decision-making processes. In multimodal vision systems, cameras with different modalities can provide complementary visual information [70]. This is particularly important, as such systems can help prevent the rapid spread of fire and its associated consequences [91]. Furthermore, by using depth-wise separated convolutions to construct a backbone layer, the number of model parameters can be reduced. One example is a lightweight convolution-based network model for developing efficient methods in the fire-protection robots [117].

A review of modern research in recent years shows that some researchers are studying firefighting conditions in space and at space bases [118,119,120]. Other studies focus on heat absorption and disruption of combustion reactions [121], fire suppression techniques used in aircraft—such as Halon 1301 and HFC-125, including in propulsion systems [121,122,123,124,125,126,127]–the design of automated fire suppression systems and robotic platforms [128,129,130,131,132,133,134], as well as various extinguishing agents and materials, with consideration of their environmental impact [135,136,137,138,139].

Modern vision techniques are also applied in fire protection in open areas, such as forests. Undoubtedly, early flame detection and real-time fire perception are critical factors that significantly contribute to mitigating the impact of wildfires, which can devastate large forest areas in a short time [22,140,141]. On the other hand, aerial patrol robots can serve as security equipment for large logistics centers, ports and docks, and public utility spaces [113,142]. An example of an unmanned aerial vehicle (UAV) equipped with an RGB-D (Red, Green, Blue, and Depth) camera and a downward-facing optical flow sensor, designed to explore indoor three-dimensional spaces, is described in [143]. In practice, unmanned aerial systems are particularly useful for the early detection of fires in locations considered too dangerous for manned aircraft or ground crews. They are also effective in providing detailed situational assessments and in the examination of natural disasters [79,105,144,145,146,147]. Specialized drones are being designed both for extinguishing wildfires and for combating flames in high-rise buildings [148,149,150]. For detection purposes, systems increasingly incorporate artificial intelligence modules that enable continuous forest monitoring and fire pixel detection [144,151,152,153]. Using such systems, it becomes possible to locate spot fires and fire lines. Through the application of computer vision, image processing techniques, and deep learning algorithms, flames can be detected with high accuracy [144,154]. Moreover, certain algorithms can also be applied to detect humans in fire environments—for example, the EDSR (Enhanced Deep Super-Resolution Network) x4-based upscaling technology [79].

Current research efforts are focused on integrating multiple factors; for instance, fire detection can now be achieved by utilizing both color features (chromatic characteristics of flames) and motion features (applying the Horn and Schunck optical flow algorithm) [155]. Fires can also be localized using the blob counter method, which counts objects in an image that are separated by a black background. A distributed control framework for multiple unmanned aerial vehicles used in wildfire tracking is currently under investigation [156], with one of the key benefits being the reduction in operational costs [141,156]. Furthermore, it is now possible to identify fire-prone conditions in advance, enabling early warnings to be issued to local populations [157]. Unsurprisingly, a growing body of research—both simulation-based and experimental—is dedicated to the use of aerial vehicles for terrain and vegetation mapping (including post-fire analysis and damage assessment), fire detection and monitoring, as well as supporting ongoing search and rescue operations [158].

An alternative method to traditional ground-based fire suppression is the use of aviation containers for extinguishing fires [159]. Other techniques include the application of fire-suppressant gases, water mist, and acoustic waves, depending on the class of fire. One component of a system in which drone and remote sensing technologies coexist is the use of fire extinguishing balls [72]. In practice, even small fire extinguishing balls can be applied to combat fires—for instance, a ball weighing approximately 0.5 kg is capable of extinguishing a grass fire with a diameter of one meter (short grass)—enabling drones to be deployed for wildfire suppression [72]. The high-pressure launch mechanism for releasing fire extinguishing bombs is described in [146]. A frequently addressed topic among researchers is the enhancement of fire suppression efficiency through the automation of water-dropping processes—specifically, determining when, at what cost, and for how long water drops should be conducted at the fire site [160]. A separate issue involves the development of water supply monitoring systems—for example, those based on LoRa wireless network technology (e.g., collecting data on water pressure levels, water levels at various nodes, etc.) [161]. Using computational methods, it is possible to carry out numerical simulations of water jet flow fields from fire nozzles to evaluate the effects of different outlet velocities and spray angles on flame suppression effectiveness [162,163]. Similar research is also being conducted on the analysis of conditions related to the refilling of onboard firefighting systems in aircraft [164].

### 3.3. Quantitative Analysis of the State-of-the-Art

As previously indicated, this section presents the results of a quantitative analysis of publications obtained from the Scopus database. The analysis is complemented by the application of a statistical procedure in the form of a chi-square test to examine the data across individual categories. The purpose of this analysis is to compare scientific publications from two periods (2015–2019 and 2020–2024) on fire protection technologies. The analysis covered 125 documents and was carried out taking into account three main categories: Document Type, Firefighting Technology, and Research Methodology. For each category, a chi-square test was performed to determine the statistical significance of the differences between the two time periods. The data in Table 3 were grouped into these categories.

**Figure 8:**
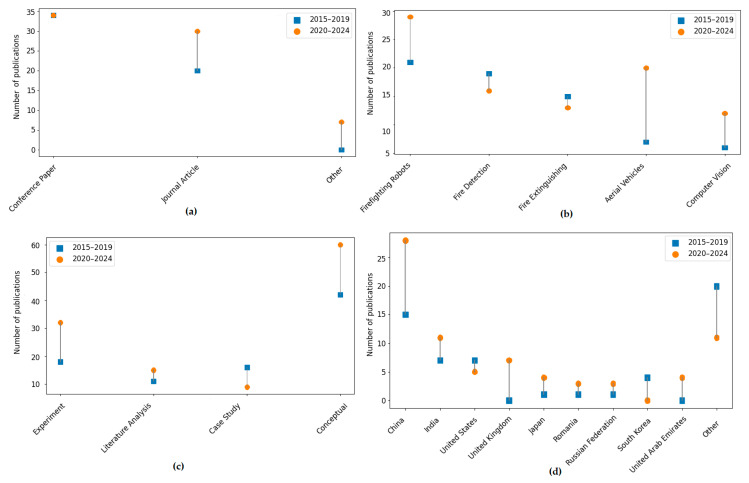
A comprehensive summary of the number of publications in the analyzed periods 2015–2019 and 2020–2024 with respect to: (**a**) the type of publication; (**b**) the technologies applied; (**c**) the adopted research methodology; and (**d**) the countries of publication.

For the category of Document Type, the result of the chi-square test is χ^2^ = 4.28, *df* = 2, *p* = 0.12, suggesting that no significant differences were observed in the distribution of document types between the analyzed periods (2015–2019 and 2020–2024). Conference publications dominate in both time periods (53.6% of all publications). For the Firefighting Technology category, the chi-square test result is χ^2^ = 7.01, *df* = 4, *p* = 0.14, suggesting that no statistically significant changes were found in this category. However, an increase in interest in aerial vehicles and vision systems can be observed from 2020 to 2024. The largest share is held by firefighting robots (40%). In turn, for the category Research Methodology, the result of the chi-square test is χ^2^ = 5.64, *df* = 3, *p* = 0.13, indicating that no statistically significant changes in the research methodology were observed. The conceptual approach continues to dominate, although the number of experiments increases during the period 2020–2024. No statistically significant differences were found in these three main categories (i.e., *p* > 0.05), indicating that the distribution of document types, technologies used, and research methodologies remained relatively stable during the analyzed periods. The largest share is held by the conceptual approach (81.6%). Despite the lack of significant differences, certain trends are visible. A comprehensive summary in the form of diagrams for all main categories (Table 3) and countries of publication (see Table 4) is provided in Figure 8.

Table 4 shows the number of scientific publications from 2015 to 2019 and 2020 to 2024, broken down by country, and their percentage share of the total. In addition, the result of the chi-square test is included, which is applied to check whether the change in the number of publications between periods is statistically significant.

As shown in Table 4, China has the highest share of publications (33%) and significant growth in the period 2020–2024 (from 15 to 28). India and the USA also show growth, but their overall share remains moderate. To sum up, the largest number of publications comes from countries other than China, India, and the USA, accounting for a 45% share. The lack of statistically significant differences means that it cannot be clearly stated that the publication activity of countries has changed in the analyzed periods. The chi-square result, χ^2^ = 2.61, *df* = 3, *p* = 0.46, means that there are no statistically significant differences in the distribution of publications between the periods 2015–2019 and 2020–2024, depending on the country. In practice, the changes in the number of publications between countries are not large enough to be considered significant (they could have occurred randomly).

Table 5 presents the number of scientific publications broken down by the type of Firefighting Technology (i.e., Firefighting Robots, Fire Detection, Fire Extinguishing, Aerial Vehicles, and Computer Vision) in the Research Methodology applied. A chi-square test is used to assess whether the observed change in the number of publications is statistically significant, considering the type of Firefighting Technology and Research Methodology.

**Figure 9:**
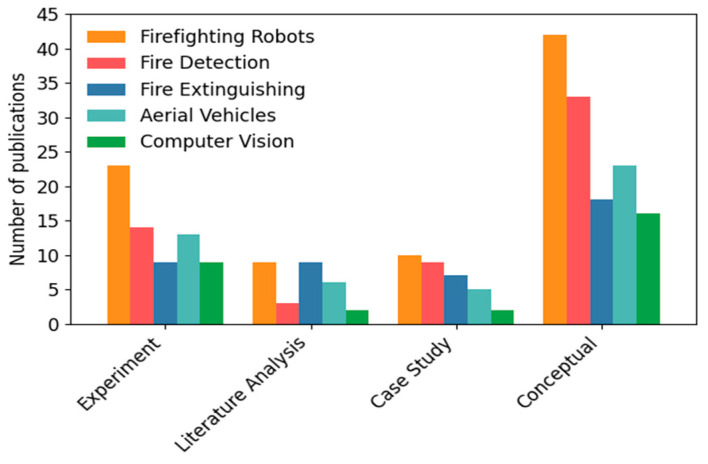
Summary of the number of publications in the Firefighting Technology category in relation to Research Methodology, broken down into subcategories within the main domain.

In practice, the subcategory Firefighting Robots dominates as a research topic, particularly in experimental and conceptual studies, while Computer Vision and Aerial Vehicles appear less frequently, but predominantly within experimental and conceptual analyses. On the other hand, the conceptual approach is the most commonly applied, appearing in as many as 102 publications (over 80%), which may indicate the early stage of development of many technologies. It can be observed that both the topics and research methods in firefighting technologies are diverse, and that there is no strong correlation between the technology type and the research method applied. A comprehensive summary of the number of publications in the Firefighting Technology category by Research Methodology is presented in Figure 9.

The results of the statistical analysis—the chi-square test, which is χ^2^ = 10.06, *df* = 12, *p* = 0.61—lead to the conclusion that the result is not statistically significant. This means that there is no clear relationship between the technology analyzed and the methodology used; the choice of research method does not differ significantly depending on the type of technology being analyzed. There is a noticeable lack of statistical significance. Although certain combinations (e.g., experiments involving robots) occur more frequently, they are not dominant enough to be considered nonrandom.

Furthermore, to quantify effect size in the chi-square test of independence, Cramér’s V coefficients as measures of association strength for each of the main categories were applied. On this basis, it becomes possible to determine the practical significance of the observed differences—that is, the strength of the associations between data points based on the obtained χ^2^ values and the size of the analyzed sample.

The results of the chi-square test, as well as the corresponding effect size measures, indicate that the observed temporal differences do not exceed the typical threshold of statistical significance within the analyzed period. However, analysis of the effect size (Cramér’s V) shows that while associations are present, they are weak in magnitude. In the case of Document Type and Research Methodology (Table 3) as well as Country (Table 4), Cramér’s V was approximately 0.18, 0.21, and 0.14, respectively, indicating weak associations. For Firefighting Technology, a small to moderate association was noted (Cramér’s V ≈ 0.24). For the cross-tabulated data in Table 5 (publications by Firefighting Technology in Research Methodology), Cramér’s V was equal to 0.16. The obtained *p*-values, combined with the relatively small Cramér’s V values for the sample, suggest the persistence of stable frameworks in the development of the research field, while simultaneously pointing to the existence of statistically unmarked trends (e.g., growing interest in UAVs and vision systems). From a practical standpoint, conference publications and conceptual works remain dominant, particularly those addressing firefighting robots—a topic that continues to be undertaken in subsequent years. Based on the percentage shares of publications in subcategories, it can be observed that although the tests did not show statistically significant differences, there is increasing interest in certain subfields, notably Aerial Vehicles (which rose from 7 to 20) and Computer Vision (which rose from 6 to 12) during 2020–2024. This may suggest a diversification of technologies that could gain substantial practical importance in the future. In this sense, it should be emphasized that the lack of statistical significance does not negate the existence of developmental directions but rather indicates that the changes are gradual and of relatively small strength within the analyzed timeframe.

### 3.4. Qualitative Analysis of Publications by Subcategories (Category Firefighting Technology)

#### 3.4.1. Firefighting Robots

This subsection discusses issues related to the use of robots in fire extinguishing [15,18,25,41,44,45,47,49,51,63,64,65,66,67,69,70,73,75,77,80,81,86,87,88,89,91,98,99,100,101,103,104,107,108,109,110,111,112,113,114,116,117,130,132,133,134,148,149,162,163].

It can be noted that some of the works are devoted directly to the technological development of robots, their application and prospects [18,63,148], their design features [25,64,65,80,99,101] and methods of protecting them at high temperatures [98], functional characteristics in case of fire [150], technical characteristics of the ground station responsible for their control [130], movement systems [133], including the ability to detect fires [41,108], remote control of robots [91], design (for example, the QRob robot for work in confined spaces [15]). While others use modeling and computational methods to find optimal movement and orientation paths in space [87,100,110], efficient interaction of individual parts of the robot [134], optimal perception of the situation in fire conditions using sensor fusion [69], control of unmanned robots [111] and tracking them [73,107,112], flame recognition by aerial robots in sunlight, reflection and bright lighting conditions [113], control of water flow supply conditions by unmanned aerial robots [162], detection of the number of victims, their condition and location [66], changing the position of the robot in space when it supplies water for fire extinguishing from a monitor nozzle [163], calculating the vertical and horizontal direction of fire spread during a fire using fire robots [114]. The authors propose a multimodal model for flame detection by a robot based on projection and attention control [70], deep learning for fire class detection and identification by an intelligent fire robot [44], a context-aware dual attention network (CG-DALNet) model to improve the performance of autonomous robots [117], a combination of a knowledge graph and a LLM (“FireRobBrain”) as the “brain” of fire robots when solving problems in dynamic conditions [116], a greedy-algorithm-based route planner to optimize robot movement in the case of multiple fires [77], and a path planning algorithm to achieve the most efficient path when the robot operates autonomously [86]. Some of the works are also devoted to the design and software of robots for their mobility and optimization of fire detection [81,132] and/or extinguishing [49,89,103,104,109,153], for their use in a group (in the Master-Slave system) with Bluetooth control to optimize fire detection and extinguishing [45], for the ability to remove obstacles on the way to a fire using a mechanical hand [47], for the creation of an IoT system using fire robots for early fire extinguishing [51], for their use in conjunction with stationary sensors in natural conditions to ensure mobility of fire detection and extinguishing [88], for movement in a fire and transmission of information about the fire using an image processing system and a communication architecture based on GSM technology and a microcontroller [67], for optimization of fire extinguishing in manual control mode using smart devices [75].

#### 3.4.2. Fire Detection

This group of publications is devoted to issues directly related to fire detection [15,17,22,26,42,43,49,52,53,55,56,57,74,76,78,80,82,83,84,88,90,96,104,106,108,127,129,131,132,145,151,154,155,157].

Thus, a general overview of current research and developments in the field of fire detection using image processing, computer vision, and deep learning is presented in [154]. The application of flame sensors and an Arduino UNO microcontroller in a fire-fighting robot is described using the Proteus 8 Professional modeling environment [80] or by controlling via Bluetooth [26,82]; robots for detecting and extinguishing fires are being developed [49,106], including those based on the Arduino UNO microcontroller [84,104,132]. Based on image analysis using machine learning methods, the possibility of creating a UAV-based forest fire extinguishing system for continuous forest monitoring and fire detection is considered [151], deep learning methods and computer vision are used [42] to quantitatively estimate the fire intensity from flame images and improve the understanding of fire calorimetry, indoor fire and smoke detection systems based on machine vision are developed using training datasets and adapted models based on the Faster R-CNN Inception V2 and SSD MobileNet V2 models [43], and in [22] an accurate intelligent YOLOv5 model based on deep learning is proposed for forest fire detection. Machine vision [145] is also used to develop an aerial sensing system (UAV) for fire detection in low visibility and smoke conditions based on multiple cameras and a thermal imaging sensor. It is proposed to perform forest fire detection based on visual perception using UAVs [155]. A learning-based approach to fuzzy smoke detection is considered for efficient and early detection of forest fires [78]. In [56], generic event trees derived from the OECD fire database are used to study the behavior and development of fires at nuclear power plants. Probabilistic analysis of fire safety in nuclear power plants, through event and fault trees supported by technical and human reliability data, is also investigated in [55]. In [129], the use of a combined scheme for tunnel fire detection based on a three-wavelength flame interval and multispectral imaging is considered, which allows determining the three-dimensional position of the fire source using camera calibration, parallax calculation, and coordinate transformation. As a component of urban tunnel fire detection, the effect of longitudinal ventilation on the system is studied using numerical modeling with FDS (Fire Dynamics Simulator) [53]. A cost-effective GSM-based fire alarm system that detects fire or smoke and sends an alarm signal to a mobile phone is proposed [76], and the detection of active forest fires and notification using a mobile application using meteorological data, fire hazard indices, and a predictive alert system [157] is considered. As an important element of fire detection, the behavior of burning fuel is studied [127] using the computational fluid dynamics (CFD) method.

The authors in [74] offer a technical description, hardware and software for an intelligent fire robot with a robotic arm that performs obstacle detection; is equipped with temperature, flame and gas sensors for fire detection and fire suppression, while it is stated that the sensors of the proposed robot are capable of detecting targets at large distances and have greater accuracy than the flame sensor installed on the QRob fire robot [15]. At the same time, QRob [15] is claimed to be more compact and capable of approaching the fire source at close distances. The fire robot [108] is also capable of detecting fire using temperature and smoke sensors. It is equipped with an LCD display that displays the temperature, smoke level, and fire intensity. The features of the heat shield, designed to protect sensors, detectors, controllers, and other components in the fire robot, are discussed in [96]. A robot with thermal and infrared cameras for fire detection and night vision is considered in [90]. Wireless networks of sensors and actors for sequential detection (sensors) and extinguishing (robots) of fires in natural conditions are proposed in [88]. Optimization of their operation is achieved through clustering, scheduling of sensor operation (sleep/active mode), and energy harvesting (EH)/motion modes for robots. A special wireless communication protocol for fire detection and fire alarm, along with a complete set of wireless automatic fire alarm systems, has been developed to achieve rapid fire detection [131]. In [57], a remote signaling and fire protection system based on the 8051 microcontroller is proposed for remote monitoring and control. A detailed description of the technical characteristics, operating principle, software, and hardware for detecting fires (flame sensor) using a fire robot is presented in [17]. The fire detection module is one of the main modules of the fire robot [83]. Information from all modules is processed by controllers, which then ensures the effective operation of the robot in detecting and extinguishing flames. The effectiveness of fire detection and extinguishing systems in a subway train is tested experimentally in [52] to study the response efficiency of these systems.

#### 3.4.3. Fire Extinguishing

In this block of publications, the main emphasis is placed on the features of Fire Extinguishing [38,39,40,48,52,54,55,57,102,104,118,119,120,121,122,123,125,126,127,128,136,137,138,139,142,149,161,164].

In [118], conceptual provisions for extinguishing fires in habitable sealed compartments of future long-term research and industrial bases located on the Moon are considered, the main fire extinguishing agent (Nitrogen) is proposed, and its required quantities are calculated, and in [119], the use of an automatic fire extinguishing system in habitable sealed compartments of an orbital station is implemented. A new technology for extinguishing fires in the habitable pressurized compartment of a manned spacecraft with simulated gravity has also been proposed [120], which consists of selecting special non-flammable polymeric materials and creating a reaction force impulse in a given direction for a certain time, which makes it possible to effectively prevent the spread of fire in conditions of long-term space flight.

The study of the flow rate of the fire extinguishing system based on HFC-125 for comparison with the fire extinguishing system based on Halon 1301 is carried out in [121], a mathematical model of the aircraft fire extinguishing system is proposed [164] and the flow characteristics of the fire extinguishing system with Halon 1301 as an extinguishing agent are studied at different filling pressures and initial temperatures. Wireless network technologies, the collection and integration of information from sensors in buildings in the fire extinguishing system are considered [161], while in [38] a method is proposed to justify the configuration of fire extinguishing systems of territorial communities in the required state, based on the conceptual and simulation models of the proposed fire extinguishing system. A new integrated system, “T-Fire”, for monitoring and extinguishing fires in trucks is proposed, operating in any conditions due to the interaction of on-board sensors with an environmentally friendly “plug and play” fire extinguishing system [39]. By modeling a fire extinguishing system with an ultrafine dry powder extinguishing agent, it was shown that the design of the helicopter engine compartment fire extinguishing system determines its discharge, flow rate, and dispersion characteristics and affects the fire extinguishing efficiency [122]. Whereas, when assessing the reliability of fire extinguishing systems in aircraft engine nacelles using modeling [123]. It was noted that simpler and shorter pipelines provide higher pressure during spraying and a higher mass flow rate of the extinguishing agent. To assess whether the halon-replacement candidate can achieve the efficiency level of Halon 1301, a fuel fire in a pallet located inside the cargo compartment of an aircraft was simulated [127] for a Halon replacement fire extinguishing system. A simulation model was employed in [126] to investigate aircraft fire extinguishing systems utilizing Halon 1301 as the extinguishing agent. The lumped parameter method [125] is employed for numerical modeling of a fire extinguishing system, with the change in freon concentration over time in the cargo compartment of an aircraft considered for the design of fire extinguishing systems. A gray, fuzzy, hierarchical mathematical model is established for high-rise buildings, and a new comprehensive assessment system is proposed for the fire risk of high-rise buildings in [149]. Modeling the spread of smoke and the evacuation of people in a library during fires, as well as researching the fire peril arrival time and evacuation situation when the fire extinguishing system fails, was conducted by the authors [142]. The technical reliability of fire detection systems, fire and smoke removal dampers, fire doors, as well as fire extinguishing systems and equipment, including fire extinguishing agent supply systems, presented in the technical documentation of nuclear power plants, is assessed based on the failure rate using Probabilistic Fire Safety Analysis [55]. In [148], the influence of ignition locations, smoke removal, and fire extinguishing systems on fire development is studied in a simulation (using FDS) of fire development on a covered pedestrian street. FDS and the coupled thermomechanical method were used [102] to study the influence of localized abrupt cooling on the temperature field, displacements, and stresses in a steel structural system of large-span buildings during a fire. A fire extinguishing calculation model based on combustion dynamics, fire physics, and fire chemistry is also considered for simulating the foam fire extinguishing process [138]. The wavelet finite element method for analyzing the distribution laws of thermal stresses, temperature, and pressure in uncoated, coated, and nanocoated liquefied petroleum gas tanks was applied in [139] to study the fire protection effect of a nanocoating on the tank. In [57], a remote signaling and fire protection system was developed using a remote terminal for monitoring the situation and for generating control outputs for fire extinguishers based on commands received from the operator of a remote-control point at a nuclear power plant.

The expansion rate of fire extinguishing foam is investigated using the similarity theory, taking into account the hydrodynamic features of the deflector-type sprinkler and the properties of the foaming solution, and a new simplified mathematical model for predicting the expansion rate is developed [128], the role of surfactants in protecting various metals and alloys commonly used as materials for fire-fighting equipment is studied [137]. The insufficient effectiveness of alcohol-resistant foams in extinguishing various polar liquids is shown by means of their experimental tests for fire extinguishing properties and tests for non-ignition [136]. The effectiveness of a high-pressure, finely atomized water fire extinguishing system for ensuring the safety of a tobacco warehouse is verified using fire tests [54]. A study was conducted on the operation of fire detection and extinguishing systems, where the systems were installed in a subway train [52], the design and characteristics of an electric vehicle fire extinguishing system were presented [40], and a prototype of a networked automated fire robot was designed and developed [104], which is equipped with a water fire extinguishing system using a spray gun and a pump.

#### 3.4.4. Aerial Vehicles

This subsection is devoted to issues related to Aerial Vehicles [46,66,68,72,78,79,97,124,126,135,140,141,143,144,145,146,147,148,151,152,153,155,156,158,159,160,164].

Thus, trends in the use of drones in firefighting are studied [148], the significant role of UAVs in extinguishing forest fires is noted [140], and the working mechanism and key stages of the implementation of a forest fire extinguishing system based on UAVs with integrated artificial intelligence capabilities are described in detail [151]. Unmanned aerial vehicles and remote sensing technologies [72] are proposed for extinguishing forest fires, where the proposed system consists of reconnaissance, communication, and fire UAVs, and extinguishing is provided by the use of fire extinguishing balls. In turn, the authors [141,156] propose a distributed control system developed for a group of UAVs, which will provide surveillance of a forest fire and enable accurate tracking of its development. An aerial sensing system in conditions of poor visibility and smoke, using machine vision for early detection of fires with a built-in intelligent processor, is presented in [145]. The detection and tracking of forest fires using UAVs is proposed to be performed using the MATLAB fuzzy toolbox and MICRODEM software [153], and a forest fire detection method using both color and motion characteristics can be used to efficiently extract and track fire pixels in aerial video sequences for application in UAV-based forest fire suppression [155]. To prioritize the actions of firefighters, it is proposed to use UAVs with a deep learning-based computer vision module [66]. And in [79], the use of autonomous drones based on the Internet of Things is considered to provide detailed situational awareness and hazard assessment for rescuers, firefighters, and police officers, as well as for fire detection and suppression. Based on visual images obtained by a UAV camera, a learning-based approach to fuzzy smoke detection is presented [78].

A coarse-to-fine framework to auto-detect wildfires that are sparse, small, and irregularly shaped, based on photo and video data obtained using UAV data, is proposed [144]. And in [143], the features of calculating a hybrid UAV flight trajectory in an unknown environment for the purpose of detecting and extinguishing a fire are considered. Optimization of the ROSMonitoring framework was performed by the authors of [68], and the results of a study including real-time monitoring of individual components of a UAV intended for extinguishing fires are presented. Design, construction, and testing of a new lightweight tethered UAV with mixed multi-rotor and water jet propulsion for forest fire fighting are presented in [158]. The design of a portable, efficient, and fast unmanned aerial vehicle for firefighting was also proposed by the authors of [146]. In [97], a multifunctional unmanned aerial vehicle with a fire detection and extinguishing function and intelligent inspection was developed. A UAV design for extinguishing fires in the open air, including in difficult weather conditions, has been implemented [46].

A simulation model has been developed [160] to calculate when, at what flow rate, and at what time it is most effective to discharge water to improve the efficiency of fire extinguishing using an aircraft. In [126], the flow characteristics of an aircraft fire extinguishing system using Halon 1301 are numerically simulated based on a simulation model. In contrast, [164] uses a mathematical model to characterize the flow rate of the fire extinguishing system. A computational fluid dynamics (CFD) method [135] is used to determine the “design concentration” of nitrogen required for fire extinguishing on an aircraft, where nitrogen is considered as a substitute for the environmentally unsafe Halon 1301. Numerical modeling of the features of the process of spraying a fire extinguishing agent in the fan compartment of a civil aircraft engine has been carried out [124], and mathematical modeling of the motion of an aircraft, an aviation fire container, and the process of using an aviation container has been performed [159]. A multi-perspective approach is proposed for a standardized methodology for modeling and analyzing a system of specialized aircraft for extinguishing forest fires [152], which will provide firefighters with a more specific and intuitive visual conceptual model of the task being performed. A theoretical justification for initially subjective, uncertain, and empirical support measures is presented within the concept of expanding the quality function of flight test support [147].

#### 3.4.5. Computer Vision

This subsection focuses on the issues related to the use of Computer Vision in fire extinguishing technologies [17,37,42,43,44,50,66,67,69,70,71,85,90,113,115,145,153,155].

A fusion approach integrating thermal imaging cameras and LiDAR sensors is studied to enhance the perception capabilities of fire robots in fire conditions by optimizing sensor calibration and further fusion [69]. A multimodal flame detection model based on projection and attention control [70] is proposed to effectively solve fire extinguishing problems by fire robots equipped with multimodal vision systems; quantitative estimation of fire severity from flame images using deep learning methods and enhanced the understanding of fire calorimetry based on visual perception using computer vision [42], while statistical and other fire data were used in [37] to probabilistically predict the location and time of fire occurrence. A spatial regression analysis model can be used to select fire hazard zones, where fire damage data is considered as the dependent variable of the spatial regression model [115].

A vision-based aerial sensing system with an embedded intelligent processor for early fire detection is considered [145]. A combination of AlexNet for fire detection and ImageNet for fire type identification is proposed for an intelligent firefighting robot, utilizing deep learning to detect, classify, and extinguish fires [44]. While an RGB image preprocessing algorithm for filtering areas suspected of being engulfed in flames and the YOLOv5 algorithm for flame recognition in the preprocessed image are used in the development of a recognition system for aerial cruise firefighting robots [113]. A method for image processing in UAVs with machine vision systems for monitoring and detecting forest fires is proposed, based on the use of color and dynamic parameters of the fire [155]. A firefighting robot equipped with a thermal imager utilizes machine vision to detect, localize, and extinguish fires in enclosed spaces [50]. The presence of an infrared camera on the robot provides night vision and the ability to record the process in real-time, while a thermal imaging camera allows for the detection of fires and the measurement of temperature [90]. A computer vision module is presented for UAVs, which contains two deep neural networks trained on images of three classes: fire, smoke, and person [66]. Meanwhile, in [153], an algorithm is developed for using a UAV video camera to detect and track forest fires, where image processing tools based on genetic fuzzy algorithms are used. The possibilities of the Internet of Things for creating a fire robot equipped with a camera for observation and spatial orientation are demonstrated [71]. An image processing system and communication architecture for a fire robot are developed, including capturing images by the robot’s camera, processing them in MATLAB, and sending them to the user via GPRS technology [67]. A machine vision-based system using surveillance camera video recordings and models based on Faster R-CNN Inception V2 and SSD MobileNet V2 models for processing them is proposed for detecting fires and smoke in buildings [43]. A fire recognition and localization system combining a convolutional neural network (CNN) with various image processing methods for recognizing and localizing fire sources in input images is presented in [70]. The design and prototype of a robot capable of identifying and avoiding obstacles, as well as detecting and extinguishing fires, are considered [17].

If we summarize the state-of-the-art, an analysis of publications from the past ten years reveals that computer vision technologies are currently being used to enhance the effectiveness and speed of fire detection and flame suppression, both indoors and in open areas (e.g., wildfires). Moreover, systems that implement deep learning techniques demonstrate strong performance in detecting fire and smoke pixels, often outperforming the range of traditional sensors due to the use of cameras (whereas conventional temperature or gas sensors have a limited detection range). A significant advantage is the faster response time; emergency services can be notified immediately, as data is processed in real time, resulting in reduced fire-related damage and an overall increase in safety. In conditions that are particularly dangerous for humans, non-flammable firefighting robots can be used (such robots can also be used to support firefighting operations carried out by firefighters and reduce the risk of them losing their lives).

It is important to note that despite substantial scientific and technological progress in the 21st century, challenges still remain. These include false alarms, a limited range of fire suppression techniques, restricted access to extinguishing agents, the need for their rapid replenishment, and the continued demand for lightweight, non-flammable structural materials. These challenges highlight the necessity for further research in the field of fire protection. The integration of multiple technologies, particularly the combination of computer vision with unmanned aerial vehicles and ground-based robots, has emerged as an innovative approach to firefighting. Once considered merely an auxiliary tool, such systems are now often a core component of fire management strategies. This integration opens up new possibilities for rapid, effective, and human-safe firefighting operations, especially in hard-to-reach locations.

## 4. Unsolved Problems

Based on the analysis of the literature, as well as a review of currently applied solutions, it can be observed that systems supporting emergency response operations remain the subject of ongoing efforts in numerous scientific and academic centers worldwide. Research typically focuses on automated robotic platforms and artificial intelligence algorithms aimed at increasing precision, as well as the accuracy and effectiveness of the actions undertaken. The results indicate that integrated data fusion systems (e.g., thermal imaging cameras and LiDAR [69,70]) and deep learning algorithms for flame detection and fire classification [42,44,66,113,144,151] are playing an increasingly important role. The significance of such solutions is confirmed by multiple studies [17,41,70], which demonstrate their benefits under various and complex conditions.

It should be emphasized that despite the advanced state of research, many open problems still remain. For example, verifying the performance of robots in real fire conditions is challenging, not only due to logistical constraints but also due to the complexity of the systems themselves, including their distributed architecture [68,88]. In practice, technical solutions based on computer vision applications enable the offloading of humans by deploying robots for firefighting tasks, which helps improve safety for firefighters during fire suppression efforts in various environments [15,22,26,39,44,45,46,47,48,49,50,51,52,53,54,55,56,57,63].

Although many of the applied solutions rely on well-established mechanisms and path-planning algorithms [85,86,87,88], there is still a lack of experimental studies validating their effectiveness in specific fire environments (e.g., during firefighting operations in high-rise buildings or industrial facilities). In practice, ongoing research focused on automated resource management represents a significant direction that directly influences the development of modern fire protection systems. On the other hand, automation also introduces certain risks—ranging from system reliability issues to real-time system interoperability.

The issue of regulatory framework and standardization for the use of fire-extinguishing robotic systems and computer vision-based systems remains unresolved. Furthermore, more in-depth research into the specifics of their calibration, maintenance, and long-term operation is also needed to assess their commercialization and ensure practical implementation on an industrial scale.

The increasing role of technologies based on artificial intelligence and process automation, which also affects the development of Fire Extinguishing Systems Based on Computer Vision (including systems, robots, approaches, and sensors), within Industry 5.0, highlights the need for a more detailed study of their environmental aspects. This applies both to their environmental friendliness and to their application in the development of green technologies [165,166], including in fire suppression. Researchers have already noted the need for eco-design of robots [167], the contribution of digital technologies [168] and robots [169] to reduce carbon emissions and harmonious economic and environmental development, as well as the need for informed political decision-making regarding their use [170]. However, in the field of fire suppression, the issue of a detailed assessment of the environmental friendliness of the robotic and artificial vision-based systems under study has not been sufficiently addressed and could be the subject of further study.

The following areas are identified as potential areas that require further research:

1. Development of integrated systems utilizing various extinguishing agents and techniques, including water mist, aerosols, and acoustic waves [10,19,20,21,135,136,137,138,139,161,162,163].
2. Design of complex multimodal systems that incorporate multiple technologies, combining data from visual cameras, thermal imaging, and conventional sensors [50,70,71,78,90].
3. Optimization of the placement of firefighting robots and systems to increase the effectiveness and response speed of fire protection measures [38,74,88].
4. Testing deep learning algorithms for flame and human detection under conditions of heavy smoke and variable lighting [79,113,117,144,155].
5. Improving techniques that integrate multispectral imaging, artificial intelligence models, and topographic mapping to enable rapid flame detection, monitoring, and effective suppression [141,144,145,146,147,156].

## 5. Conclusions

Despite decades of research, there has been a significant increase in publications on the issue under study only in the last 10 years. The leading countries in terms of the number of publications are China, the United States, and India. Analysis of the studies revealed the distribution of keywords into 4 clusters: “Fire extinguishers”, “Fire protection”, “Robots”, and “Firefighting equipment”. The general trend is a shift in research to the technical side related to machine learning methods and robotics.

Progress is being made in research aimed at the technical development of fire-fighting robots, the improvement of software and hardware, the development of artificial intelligence and the Internet of Things in fire-fighting technologies, as well as the significant use of computational methods and modeling in assessing the characteristics of fire-fighting equipment or its interaction with the environment. Further research has shown that automated firefighting systems, as well as technologies based on artificial intelligence and computer vision, are playing an increasingly important role in modern fire protection. The synergy of various technical solutions—from communication systems and the integration of various sensors (both smart and traditional) to the implementation of algorithms, deep learning methods, and fire suppression techniques—promotes more effective flame detection and fire suppression in both enclosed and open spaces.

Despite significant advancements over the past decade, a number of research challenges remain—such as verifying the effectiveness of individual solutions under real (non-simulated) fire conditions, optimizing the placement of robots and firefighting systems, and improving system reliability and interoperability. It is expected that future work will focus on the development of collaborative systems that integrate several complementary or interacting detection and extinguishing techniques, depending on the fire’s class, size, and characteristics. This will be made possible through the implementation of modern solutions based on continuous data analysis and the automation of decision-making processes.

## Figures and Tables

**Table 1:** Top 5 author affiliations.

Affiliation	Number of Publications
University of Science and Technology of China	42
Ministry of Education of the People’s Republic of China	20
Technion–Israel Institute of Technology	20
Trinity College, Hartford, U.S.	18
National Institute of Standards and Technology, U.S.	16

**Table 2:** Top 5 authors.

Author	Number of Publications
Ahlgren D.J.	19
Anon	17
Verner I.M.	17
Zhang J.	12
Tadokoro S.	11

**Table 3:** Publications by year in all categories.

Name	2015–2019	2020–2024	All Years	Share [%]	Chi-Square
**Total**	54	71	125	100.0	χ^2^
**Document Type**					
Conference paper	33	34	67	53.6	χ^2^ = 4.28 (*df* = 2, *p* = 0.12)
Journal article	20	30	50	40.0	
Other ^a^	1	7	8	6.4	
**Firefighting Technology** ^b^					
Firefighting Robots	21	29	50	40.0	χ^2^ = 7.01 (*df* = 4, *p* = 0.14)
Fire Detection	19	16	35	28.0	
Fire Extinguishing	15	13	28	22.4	
Aerial Vehicles	7	20	27	21.6	
Computer Vision	6	12	18	14.4	
**Research Methodology** ^c^					
Experiment	18	32	50	40.0	χ^2^ = 5.64 (*df* = 3, *p* = 0.13)
Literature Analysis	11	15	26	20.8	
Case Study	16	9	25	20.0	
Conceptual	42	60	102	81.6	

^a^ In this case, other documents are understood to refer to book chapters and reviews. ^b^ In practice, each document can be classified into more than one technology category if it thematically falls within more than one of the analyzed subdomains. ^c^ In practice, more than one research method is assigned to each document if it involves the application of multiple research methodologies.

**Table 4:** Publications by year in countries.

Name	2015–2019	2020–2024	All Years	Share [%]	Chi-Square
**Total**	56	76	132	100.0	χ^2^
**Country**					
China	15	28	43	0.33	χ^2^ = 2.61 (*df* = 3, *p* = 0.46)
India	7	11	18	0.14	
USA	7	5	12	0.09	
Other ^a^	27	11	59	0.45	

^a^ Apart from the countries listed in Table 4, the highest publication activity was observed in the United Kingdom, Japan, Romania, the Russian Federation, South Korea, and the United Arab Emirates (see Figure 8d).

**Table 5:** Publications by Firefighting Technology in Research Methodology.

Name	Firefighting Robots	Fire Detection	Fire Extinguishing	Aerial Vehicles	Computer Vision	Total	Chi-Square
**Total**	50	35	28	27	18	125	χ^2^
**Research Methodology**							
Experiment	23	14	9	13	9	50	χ^2^ = 10.06 (*df* = 12, *p* = 0.61)
Literature Analysis	9	3	9	6	2	26	
Case Study	10	9	7	5	2	25	
Conceptual	42	33	18	23	16	102	

## Data Availability

Not applicable.
